# Mapping Quantitative Trait Loci onto Chromosome-Scale Pseudomolecules in Flax

**DOI:** 10.3390/mps3020028

**Published:** 2020-04-04

**Authors:** Frank M. You, Sylvie Cloutier

**Affiliations:** Ottawa Research and Development Centre, Agriculture and Agri-Food Canada, Ottawa, ON K1A 0C6, Canada; sylviej.cloutier@canada.ca

**Keywords:** flax, association mapping, genome-wide association study (GWAS), simple sequence repeat (SSR), single nucleotide polymorphism (SNP), quantitative trait loci (QTL), chromosome-scale pseudomolecules

## Abstract

Quantitative trait loci (QTL) are genomic regions associated with phenotype variation of quantitative traits. To date, a total of 313 QTL for 31 quantitative traits have been reported in 14 studies on flax. Of these, 200 QTL from 12 studies were identified based on genetic maps, the scaffold sequences, or the pre-released chromosome-scale pseudomolecules. Molecular markers for QTL identification differed across studies but the most used ones were simple sequence repeats (SSRs) or single nucleotide polymorphisms (SNPs). To uniquely map the SSR and SNP markers from different references onto the recently released chromosome-scale pseudomolecules, methods with several scripts and database files were developed to locate PCR- and SNP-based markers onto the same reference, co-locate QTL, and scan genome-wide candidate genes. Using these methods, 195 out of 200 QTL were successfully sorted onto the 15 flax chromosomes and grouped into 133 co-located QTL clusters; the candidate genes that co-located with these QTL clusters were also predicted. The methods and tools presented in this article facilitate marker re-mapping to a new reference, genome-wide QTL analysis, candidate gene scanning, and breeding applications in flax and other crops.

## 1. Introduction

Most traits of importance in plant breeding are quantitative and controlled by polygenes with minor effects on phenotypes. Traditional quantitative genetics can estimate overall genetic effects or variances of polygenes for quantitative traits through dedicated genetic designs [1], providing a theoretical guide for plant breeding. With the development of molecular markers and high-throughput genotyping techniques, individual polygenic loci on chromosomes and their effects on phenotypes can be detected and estimated using statistical genomics approaches. Such polygenic loci on chromosomes are called quantitative trait loci (QTL). They are associated with phenotype variation of quantitative traits and are usually mapped in various populations using molecular markers such as simple sequence repeats (SSRs) or single nucleotide polymorphisms (SNPs). Generally, QTL can be identified by two main approaches: linkage mapping (LM) and association mapping (AM) or genome-wide association study (GWAS) [2]. LM uses bi-parental populations, such as F_2_, recombinant inbred line (RIL), doubled haploid (DH), and backcross (BC) populations, to identify loci responsible for trait variation between parents based on recombination-based genetic linkage maps [3]. AM relies on linkage disequilibrium (LD) between markers and QTL. AM uses a more diverse genetic panel to overcome the phenotypic diversity limitation of bi-parental populations. This diversity limitation may include natural germplasm collections, or, more often, panels including germplasm accessions and breeding lines, or multi-parent populations such as nested association mapping (NAM) [4,5,6] and multi-parent advanced generation intercross (MAGIC) populations [7,8,9,10]. QTL can be exploited for gene cloning, marker-assisted breeding, and genomic selection or prediction.

Cultivated flax (*Linum usitatissimum* L.) is a self-pollinating annual crop valued for its seed oil and stem fibre. Phenotypic selection remains a major conventional breeding approach to improve traits of agronomic importance in flax. To accelerate the application of molecular breeding, a large number of molecular markers [11,12,13,14] and genetic populations [15,16,17,18] have been developed to assist QTL identification in the last decade. Using these genetic resources, a total of 313 QTL for 31 traits (13 seed yield and agronomic traits, 11 seed quality traits, four fibre traits, and three disease resistance traits) were reported in 14 studies (Table 1 and Table 2). These QTL were identified mainly using SSR or SNP markers with LM or AM/GWAS (Table 2). The studies using LM were based on genetic maps [15,18,19,20,21,22,23,24], while those using AM or GWAS were based on the flax scaffold sequences [17,25,26], the early (hereafter pre-released) version of chromosome-scale pseudomolecules (PCPs) [27,28] or the most recent release of the chromosome-scale pseudomolecules (RCPs) [14,29] (Table 2). The use of different references in the QTL identification studies made it difficult to compare the results across studies, genome-wide QTL analysis, candidate gene prediction, and breeding applications. Thus, the objectives of this study were to develop methods and corresponding software tools to uniquely map the QTL identified in different studies onto the RCPs [29]. These methods and tools were designed to be applicable to studies in flax as well as other crops.

## 2. Materials and Methods

### 2.1. The Most Recent Release of the Chromosome-Scale Pseudomolecules

The chromosome-scale pseudomolecules for flax were recently released [29]. A total of 622 scaffolds from the flax reference genome [25] were sorted onto 15 chromosomes, totalling 316.2 Mb. Thus, the SNPs identified based on the scaffold reference sequences can be accurately mapped to the pseudomolecules. The 15 pseudomolecule sequences corresponding to 15 chromosomes were downloaded from the National Center for Biotechnology Information (NCBI) database. The accession numbers of the pseudomolecules for the 15 chromosomes are CP027619 (Lu1), CP027626 (Lu2), CP027627 (Lu3), CP027628 (Lu4), CP027629 (Lu5), CP027630 (Lu6), CP027631 (Lu7), CP027632 (Lu8), CP027633 (Lu9), CP027620 (Lu10), CP027621 (Lu11), CP027622 (Lu12), CP027623 (Lu13), CP027624 (Lu14), and CP027625 (Lu15). The chromosome sizes are listed in Table S1.

### 2.2. Marker Infomation of QTL in Flax

All 313 flax QTL reported in the 14 studies (Table 2) were identified from three types of markers: amplified fragment length polymorphisms (AFLPs), SSRs, and SNPs. PCR primer sequences of AFLPs and SSRs were retrieved from the literature [15,19,20,21,23,24]. For the SNPs named based on the scaffold sequences, their scaffold names and coordinates were collected directly from the publications [17,26]. For the SNPs identified without a reference [18], flanking sequences of the SNP markers were downloaded from the publication [18]. All available primer sequences of SSR markers and flanking sequences of SNP markers for the identified QTL are listed in Tables S2 and S3, respectively.

### 2.3. Mapping PCR-Based Markers to the Most Recent Release of the Chromosome-Scale Pseudomolecules

PCR primer sequences of markers were mapped onto the RCPs using the electronic PCR (E-PCR) tool [31]. A pipeline using E-PCR was developed. This pipeline includes two Perl scripts: ProgramS1_prepare_rePCR.pl (Program S1) and ProgramS2_rePCR_pipeline.pl (Program S2). Program S1 is a script that creates a search database of the RCPs, outputting two files for the downstream analysis: *.famap and *.hash. Program S2 is a script that performs electronic PCR to map paired primers onto the RCPs, generating result files with coordinates of the primers on chromosomes and their amplicon sizes. No nucleotide mismatches or gaps were allowed. The instructions of these programs are described in User guide S1.

PCR primers designed from sequences of different genotypes could not always be accurately mapped to the RCPs using the E-PCR approach. In such cases, BLASTN searches were performed to ascertain their map positions.

### 2.4. Mapping SNPs to the Most Recent Release of the Chromosome-Scale Pseudomolecules

If SNPs are identified using the flax scaffold sequences [25], their coordinates can be accurately converted to the RCPs’ coordinates. The Perl script ProgramS3_convert_scaffold_coordinates_to_pseudochr.pl (Program S3) executes this conversion. A database file for the accurate relationship between the scaffolds and the RCPs (Table S4) is required to run this program. The instructions of this script are described in User guide S1.

For the SNPs identified without a reference sequence [18], the flanking sequences of the SNPs were searched against the RCPs using BLASTN at an E-value of 10^−30.^ The alignment regions of top hits were used and manually verified.

For the SNPs based on the PCPs in two publications [27,28], their scaffold names and corresponding coordinates on the scaffolds were retrieved from the raw SNP data as these SNPs were initially identified from the scaffolds, followed by conversion to the RCPs using Program S3.

### 2.5. Grouping QTL to Clusters

QTL mapping software tools can detect multiple quantitative trait nucleotides (QTNs) from a small region that may be grouped into the same QTL or a QTN cluster based on the LD between markers [14]. QTNs detected in different populations cannot be grouped based on population-dependent marker LD. To provide a simple solution, we opted to group in a single QTL cluster all QTL located within a 200 kb window covering the 100 kb upstream and 100 kb downstream regions of the QTN position.

### 2.6. Candidate Gene Analysis Based on the Most Recent Release of the Chromosome-Scale Pseudomolecules

As the RCPs [29] were generated by sorting and refining the existing scaffold sequences [25], no changes were made to the original gene annotations on the scaffold sequences. However, the new coordinates of these genes on the RCPs were not previously released [29]. The RCPs contain 42,277 protein coding genes, of which 1,327 were predicted to be resistance gene analogs (RGAs) [29]. To facilitate genome-wide candidate gene analyses, the revised version of the script “ProgramS3_convert_scaffold_coordinates_to_pseudochr.pl” was used to convert the coordinates of the genes on the scaffolds onto the RCPs. All genes and RGAs and their coordinates on the RCPs are listed in Tables S5 and S6, respectively. These genes were mapped to orthologous genes of the model species *Arabidopsis thaliana* using BLASTP of flax protein sequences against *A. thaliana* protein sequences at an E-value of 10^−10^. A total of 15,323 unique *A. thaliana* genes were mapped. Then, the flax genes were searched against the NCBI non-redundant protein database (nr) at an E-value of 10^−5^, and functional annotations were generated using a custom script that integrates protein annotation information of top hits and the orthologous *A. thaliana* genes. The annotation results were added to the gene list. A genome-wide gene scan along chromosomes for QTL was performed to characterize the underlying genomic regions and identify candidate genes. The genes within a 200-kb window covering the 100 kb upstream and downstream regions of the QTN position were scanned. A Perl script ProgramS4_flax_QTL_candidate_gene_scanning.pl was developed (Program S4) to scan potential candidate genes for given QTL based on the gene annotation database files in Table S5 (for all protein coding genes) and Table S6 (for RGAs only). The instructions for this program are described in User guide S1.

## 3. Results

### 3.1. Mapping QTL onto the Most Recent Release of the Chromosome-Scale Pseudomolecules

**Figure 1:**
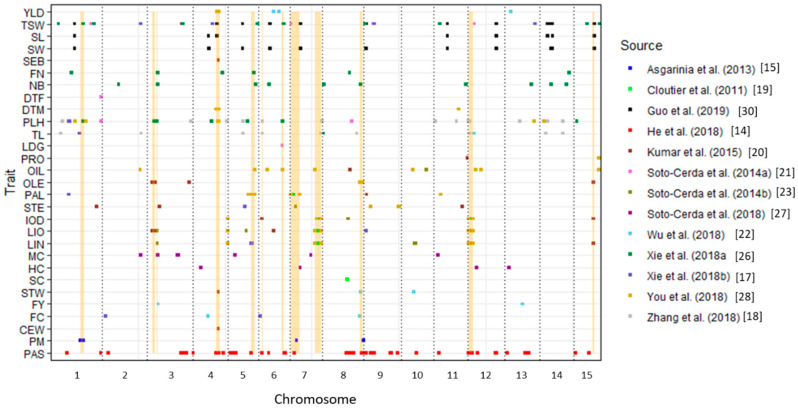
Distribution of 308 QTL associated with 29 traits mapped onto flax chromosomes. Of these QTL, 67 for pasmo resistance and 46 for thousand-seed weight, seed width and seed length have been previously mapped on the most recent release of the flax chromosome-scale pseudomolecules [14,30]. Two fusarium wilt QTL [24] were not included because of incomplete information. Co-located regions are highlighted in yellow. See Table 1 for the trait name abbreviations.

In all 14 publications reporting flax QTL, only 67 newly reported pasmo QTL and 46 QTNs associated with seed length, seed weight and 1000-seed weight were based on the RCPs [14,30]. Therefore, the mapping of the remaining 200 QTL onto the RCPs was performed. A total of 195 QTL uniquely mapped to the RCPs of 15 chromosomes, including 40 SSRs and 36 SNPs from genetic maps, 75 SNPs from the scaffolds, and 44 SNPs from the PCPs (Figure 1 and Table 3). Markers *afB13* and *afXR6* for two powdery mildew QTL were not mapped because their AFLP primer sequences were not available [24]. One QTL for branching score failed to map because its SSR marker *Lu2067a* could not be mapped to any region on the RCPs; this was likely because the marker was designed from a genotype different from the reference genome (cv CDC Bethune). Finally, the marker *Lu8_185009* for QTL *uq.C8–2* associated with plant height (PLH) and technical length (TL) [18] mapped to two different chromosomes (Chr 4 and Chr 7).

It is important to pinpoint that the SSRs/SNPs corresponding to a single marker or a pair of flanking markers from genetic maps were mapped to a genomic region on a pseudomolecule, while the SNPs from the scaffold sequences or the PCPs were anchored exclusively to single nucleotide positions representing their QTL peak locations.

### 3.2. Identical or Co-Located QTL

QTL that mapped to the same RCPs were comparable across studies, mapping populations, and traits. Based on the 200 kb upstream and downstream region rule, the 195 QTL/markers for the 26 traits mapped to the RCPs were grouped into 133 QTL clusters (Table 3). The QTL with the same numbers in the “Co-location” column in Table 3 were deemed to belong to the same QTL clusters, indicating identical or co-located QTL. QTL for 16 of the 29 traits were identified in two or more studies, of which 12 had one or more QTL located at the same positions or within the same QTL clusters (Table 1), thereby supporting the accuracy of the QTL through validation across studies.

Some QTL were validated in several studies that differed in marker types (SSRs or SNPs), populations (bi-parental population or diverse germplasm panel), or statistical methods used for QTL mapping (Table 1 and Table 2). For example, *QTL-195* (*QDTM-Lu4.1*) and *QTL-54* (*QDm.BM.crc-LG4*) on Chr 4 corresponded to the same QTL for days to maturity (DTM) identified in two different studies [20,28]. *QTL-187* (*QIOD-Lu7.2*) and *QTL-7* (*QIod.crc-LG7*) on Chr 7 for iodine value (IOD) [19,28], *QTL-190* (*QLIN-Lu7.2*) and *QTL-5* (*QLin.crc-LG7*) on Chr 7 for linolenic acid content (LIN) [19,28], *QTL-6* (*QLin.crc-LG16*) and *QTL-33* (*QLin-LG12.3*) on Chr 12 for LIN [19,23], and *QTL-4* (*QLio.crc-LG16*) and *QTL-30* (*QLio-LG12.3*) on Chr 12 for linoleic acid content (LIO) [19,23] were additional examples of the same QTL identified in different studies. Some QTL or QTNs were grouped into single QTL because their coordinates on chromosomes were close or identical and, historical recombinations may not have been present in the population; for example, *QTL-144* (*scaffold11-96400*) and *QTL-145* (*scaffold11-96569*) on Chr 1 for steric acid content (STE) [17], and *QTL-155* (*scaffold297-275131*), *QTL-100* (*scaffold297_275113*), and *QTL-154* (*scaffold297-275113*) on Chr 1 for technical length (TL) corresponded to unique QTL (Co-location cluster No. 46 and 9 in Table 3) [17,26].

Some co-located QTL may lead to their pleiotropic effects on multiple traits. Thirteen genomic regions that had at least three identical or co-located QTL were observed (yellow highlights in Figure 1 and Table 3). For example, eight QTL—*QTL-195* (*QDTM-Lu4.1*), *QTL-168* (*QYLD-Lu4.1*), *QTL-179* (*QPLH-Lu4.3*), *QTL-49* (*QCw.BM.crc-LG4*), *QTL-54* (*QDm.BM.crc-LG4*), *QTL-52* (*QSpb.BM.crc-LG4*), *QTL-50* (*QSw.BM.crc-LG4*), and *QTL-53* (*QYld.BM.crc-LG4*)—were co-located between positions 13,170,489 and 15,040,682 bp on Chr 4 and had pleiotropic effects on phenotypes of six traits: DTM, YLD, PLH, cell wall content (%) (CEW), seeds per boll (SEB), and straw weight (STW). Thus, this is an important genomic region controlling seed yield and related agronomic traits. As noted and discussed previously [19,20,28], *QTL-186* (*QIOD-Lu4.1*), *QTL-189* (*LIN-Lu4.1*), and *QTL-192* (*QLIO-Lu4.1*) were co-located at position 19,907,982 bp on Chr 4; *QTL-193* (*QLIO-Lu7.2*), *QTL-190* (*QLIN-Lu7.2*), *QTL-187* (*QIOD-Lu7.2*), *QTL-7* (*QIod.crc-LG7*), *QTL-5* (*QLin.crc-LG7*), and *QTL-3* (*QLio.crc-LG7*) were between positions 14,540,252 and 17,976,903 bp on Chr 7; *QTL-188* (*QIOD-Lu12.3*), *QTL-191* (*QLIN-Lu12.3*), and *QTL-194* (*QLIO-Lu12.3*) located in the 489,561 and 2,981,562 bp interval on Chr 12; and *QTL-6* (*QLin.crc-LG16*), *QTL-33* (*QLin-LG12.3*), *QTL-4* (*QLio.crc-LG16*), *QTL-30* (*QLio-LG12.3*), and *QTL-8* (*QIod.crc-LG16*) positioned between 2,036,216 and 3,802,807 bp on Chr 12. These four genomic regions contributed greatly to the genetic variation for LIO, LIN, and IOD in several flax populations [19,20,28].

### 3.3. Candidate Genes for QTL

The resolution of current QTL mapping or GWAS technologies is insufficient to pin QTL to accurate locations of genes or genetic features controlling traits. A simple approach for predicting candidate genes is to investigate the annotated genes in the vicinity of QTL, such as a window of 200 kb flanking the QTL [14,20]. Our ability to position most of the previously reported QTL to the RCPs makes it possible to perform an overall genome-wide candidate gene scan along chromosomes. Thus, all potential candidate genes of the 195 QTL listed in Table 3 were scanned. A total of 7,821 unique candidate genes co-located with the 133 QTL clusters (Table S7). These candidate genes can be further analysed and validated. For example, three QTL for powdery mildew resistance were identified [15] and mapped to chromosomes 1, 7, and 9 (Table 3, Figure 1). Some RGAs were found in the vicinity of the QTL, i.e., within the pre-defined window (Table 4). One nucleotide-binding-site (NBS) encoding gene (*Lus10026765*), one transmembrane coiled-coil (TM-CC) gene (*Lus10023437*), and several receptor-like protein kinase (RLK) genes co-located with these QTL.

## 4. Discussion

The RCPs, representing the first chromosome-scale flax reference sequence, were released to the NCBI database in 2018 [29]. This new flax genome reference has previously been adopted for genomic studies, such as QTL identification. Prior to this release, many QTL had been identified based on different reference sequence versions (Table 2); thus, it is necessary to re-map these QTL onto the most recent and comprehensive flax reference (RCP). In addition, some research groups have already adopted the scaffold-based reference to identify SNPs and have performed other genomic studies. Consequently, more current methods and software tools are required for this re-mapping. For this purpose, we developed several utility tools, including scripts for mapping PCR- and SNP-based QTL onto the RCPs, grouping QTL in terms of a predefined window size, and performing genome-wide candidate gene analysis. These tools were successfully used to map 195 out of 200 QTL onto the new reference. Only five QTL failed to map because of incomplete information. This demonstrates the reliability and robustness of the methods, especially those for mapping the scaffold-based SNPs to the new reference, which is unique to this study. No other methods were available because this conversion must be based on the accurate coordinates of the scaffolds on pseudomolecules that were generated by the authors of this article [29]. The QTL positioned onto the RCPs and their gene candidates can be further validated and analysed on a genome-wide basis. Comparability across different studies and genetic populations will facilitate their further evaluation for applications in flax breeding.

The methods and the computer scripts described here are not only suitable for flax, but are also applicable to other crops. In wheat, for example, a large number of PCR- and SNP-based markers have been developed from different genetic maps and many versions of reference sequences, which are deposited in genome databases such as GrainGenes (https://wheat.pw.usda.gov/GG3/) and T3/Wheat (https://triticeaetoolbox.org/wheat/). However, the first version of the chromosome-based reference sequence (RefSeq v1.0) was just recently released by the International Wheat Genome Sequencing Consortium [32]. Thus, the re-mapping of existing markers onto the new wheat reference necessitates software tools. Program S1 and Program S2, which adopted the widely accepted E-PCR tool [31] to map PCR primers to a reference, can be directly used for the mapping of the existing PCR-based markers to the new reference. In addition, the basic methodology of Program S3 and Program S4 is useful for the development of new tools specifically based on the wheat reference and gene annotation databases.

It is noteworthy that the gene annotation information of the new flax reference was not available in the NCBI or in any other databases or publications. Although being reported through personal communications, this is the first release of the complete gene annotation of the chromosome-scale flax reference (Table S4). This information is presented in addition to the flax reference [29] to facilitate genome-wide candidate gene analysis of QTL along chromosomes and other genomic studies. The RGAs, a subset of the flax genes (Table S6), are also useful for candidate gene prediction of disease resistance QTL.

## 5. Conclusions

This article details the methods, software tools, and database files developed to uniquely map the QTL previously identified from different references onto the RCPs. The methodology can be used not only for flax, but also for other crops. Using the methodology described here, 195 out of 200 PCR- and SNP-based QTL markers that were not based on the RCPs were successfully sorted into the 15 chromosomes of the RCPs and grouped into 133 co-located QTL clusters, thereby demonstrating genomic regions associated with, and/or pleiotropic to, important agronomic and seed quality traits. These re-mapped chromosome-based QTL can be easily compared across studies and facilitate genome-wide QTL analysis, candidate gene prediction, and further validation for breeding applications.

## Figures and Tables

**Table 1:** Number of QTL associated with 31 traits in flax.

Category	No	Trait	Abbreviation	Total QTL Identified	Total Unique QTL	Source
Seed yield and agronomic traits	1	Seed yield	YLD	5	4	[20,22,28]
	2	Thousand seed weight (g)	TSW	45	44	[17,21,22,26,30]
	3	Seed length (mm)	SL	10	10	[30]
	4	Seed width (mm)	SW	15	15	[30]
	5	Seeds per boll	SEB	1	1	[20]
	6	Fruit (boll) number	FN	9	8	[17,26]
	7	Branching score	BSC	1	1	[21]
	8	Number of branches	NB	13	13	[26]
	9	Days to flowering	DTF	1	1	[21]
	10	Days to maturity	DTM	3	2	[20,28]
	11	Plant height (cm)	PLH	33	30	[18,21,22,26,28]
	12	Technical length (cm)	TL	17	13	[17,18,22,26]
	13	Lodging	LDG	2	1	[21]
Seed quality	14	Iodine value	IOD	8	7	[19,20,23,28]
	15	Protein content (%)	PRO	2	2	[20,28]
	16	Oil content (%)	OIL	10	10	[20,23,28]
	17	Oleic (%)	OLE	4	4	[20,28]
	18	Palmitic (%)	PAL	7	5	[17,19,20,28]
	19	Stearic (%)	STE	8	7	[17,20,23,28]
	20	Linoleic (%)	LIO	11	9	[17,19,20,23,28]
	21	Linolenic (%)	LIN	12	10	[17,19,20,23,28]
	22	Seed mucilage content	MC	7	7	[27]
	23	Seed hull content	HC	4	4	[27]
	24	Seed colour	SC	2	1	[19]
Fibre	25	Straw weight (g)	STW	4	4	[20,22]
	26	Fibre yield (g)	FY	2	2	[22]
	27	Fibre content (%)	FC	4	4	[17,22]
	28	Cell walls (%)	CEW	1	1	[20]
Disease	29	Fusarium wilt rating	FW	2	2	[24]
	30	Powdery mildew rating	PM	3	3	[15]
	31	Pasmo rating	PAS	67	67	[14]

**Table 2:** QTL identification studies in flax.

Population	Pop Size	Markers	Method ^1^	Ref ^2^	Total QTL	No. of QTL Identified/Trait ^3^	Source
DH	59	8 RFLPs, 213 AFLPs	LM	GM	2	2/FW	[24]
DH	78	113 SSRs, 5 SNPs, 4 genes	LM	GM	9	2/LIO, LIN, IOD; 1/PAL; 2/SC	[19]
F3-F4	300	143 SSRs	LM	GM	3	3/PM	[15]
Core collection	390	464 SSRs	AM	GM	11	5/TSW; 1/DTF; 2/PLH; 1/BSC; 2/LDG	[21]
Core collection	390	460 SSRs	AM	GM	9	1/OIL; 1/STE; 3/LIO; 3/LIN; 1/IOD	[23]
RIL	243	329 SNPs, 362 SSRs	LM	GM	20	1/PAL; 3/STE; 3/OLE;2/LIO; 1/LIN; 2/IOD; 1/OIL; 1/PRO; 1/CEW; 1/STW; 1/TSW; 1/SEB; 1/YLD; 1/DTM	[20]
2 RILs	233	4,497 SNPs	LM	GM	24	14/PLH; 10/TL	[18]
F2	112	2,339 SNPs	LM	GM	12	1/PLH; 1/TL; 3/YLD; 3/STW; 2/FY; 2/FC	[22]
Core collection	224	146,959 SNPs	AM	SS	43	9/PLH; 3/TL; 13/NB; 8/FN; 10/TSW	[26]
Core collection	224	584,987 SNPs	AM	SS	23	2/PLH; 1/FN; 8/TSW; 3/TL; 1/PAL; 2/STE; 1/LIO; 3/LIN; 2/FC	[17]
Core collection	200	771,914 SNPs	AM	PCPs	11	7/MC; 4/HC	[27]
2 RILs and 1 DH	260	17,288 SNPs	AM	PCPs	33	1/YLD; 8/OIL; 5/PLH; 4/PAL; 3/IOD, LIN, LIO, 2/DTM; 2/STE; 1/PRO; 1/OLE	[28]
Core collection	370	258,873 SNPs	AM	RCPs	67	67 PAS	[14]
Germplasm collection	200	674,074 SNPs	AM	RCPs	46	10/SL; 15/SW; 21/TSW	[30]

Pop: population. Ref: reference sequences or linkage maps for QTL identification. ^1^ LM: bi-parental population-based QTL mapping; AM: association mapping or genome-wide association study. ^2^ GM: genetic map; SS: scaffold-based reference sequences [25]; RCPs: recent release of the chromosome-scale pseudomolecules [29]; PCPs: pre-released version of the chromosome-scale pseudomolecules. ^3^ See Table 1 for trait name abbreviations.

**Table 3:** QTL mapped to the recently released chromosome-scale pseudomolecules.

QTL No	Trait	*QTL/Marker ID*	LG/Scaffold	Flanking Markers	Chr	Coordinates on chr	Co-Location	Source
1	FW	*afB13*	6	*afB13*	NA	NA	NA	[24]
2		*afXR6*	10	*afXR6*	NA	NA	NA	
3	LIO	*QLio.crc-LG7*	7	*FAD3A/Lu44E4*	7	16089395-16092602	70	[19]
4		*QLio.crc-LG16*	16	*Lu206-Lu765B*	12	2036216-2041030	109	
5	LIN	*QLin.crc-LG7*	7	*FAD3A/Lu44E4*	7	16089395-16092602	70	
6		*QLin.crc-LG16*	16	*Lu206-Lu765B*	12	2036216-2041030	109	
7	IOD	*QIod.crc-LG7*	7	*FAD3A/Lu44E4*	7	16089395-16092602	70	
8		*QIod.crc-LG16*	16	*Lu206-Lu765B*	12	2038322-2038517	109	
9	PAL	*QPal.crc-LG9*	9	*Lu741-Lu675*	7	1518897-2017169	66	
10	SC	QL*.crc-LG22	22	*Colour-Lu178*	8	14838877-14839100	75	
11		Qb*.crc-LG22	22	*Colour-Lu178*	8	14838877-14839100	75	
12	PM	*QPM-crc-LG1*	1	*Lu2698-Lu2712*	1	16920407-18739647	11	[15]
13		*QPM-crc-LG7*	7	*Lu2810-Lu2832*	7	3817603-3817863	66	
14		*QPM-crc-LG9*	9	*Lu1125a-Lu932*	9	357191-357510	83	
15	TSW		3	*Lu2164*	1	22948222-22948580	13	[21]
16			6	*Lu2555*	6	14948801-14948986	65	
17			7	*Lu2532*	7	661757-662020	66	
18			7	*Lu58a*	12	3802629-3802807	111	
19			9	*Lu526*	9	5936422-5936694	88	
20	DTF		1	*Lu943*	1	28800644-28800902	16	
21	PLH		1	*Lu943*	1	28800644-28800902	16	
22				*Lu316*	8	17106045-17106266	79	
23	BSC		22	*Lu2067a*	NA		NA	
24	LDG		6	*Lu2560*	6	13553559-13553779	63	
25			6	*Lu2564*	6	13620999-13621234	63	
26	OIL	*QOil-LG9.1*	9	*c31-s67_Lu181*	10	14217309-14219605	95	[23]
27	STE	*QSte-LG7.1*	7	*c175-s1216_Lu146*	7	3308199-3308517	66	
28	LIO	*QLio-LG3.1*	3	*c729-s156_Lu3262*	3	6080016-6080189	24	
29		*QLio-LG5.2*	5	*c30-s11_Lu164*	5	10600927-10601125	47	
30		*QLio-LG12.3*	12	*c306-s98_Lu765B*	12	2036216-2041030	109	
31	LIN	*QLin-LG3.1*	3	*c729-s156_Lu3262*	3	6080016-6080189	24	
32		*QLin-LG5.2*	5	*c202-s39_Lu41*	10	7602629-8066018	94	
33		*QLin-LG12.3*	12	*c306-s98_Lu765B*	12	2036216-2041030	109	
34	IOD	*QIod-LG8.1*	8	*c46-s505_Lu2102*	8	15166626-15166926	76	
35	PAL	*QPal.BM.crc-LG7*	7	*Lu402/Lu7-1820805*	9	2026186-2026487	86	[20]
36	STE	*QSte.BM.crc-LG1*	1	*Lu2183a/Lu1-2670961*	1	26435050-26435329	15	
37		*QSte.BM.crc-LG3*	3	*Lu3-8415336/Lu2164*	3	7263087	28	
38		*QSte.BM.crc-LG11*	11	*Lu2128/Lu11-19000928*	11	16797707-16797907	102	
39	OLE	*QOle.BM.crc-LG3-1*	3	*Lu3-3979616/Lu3-5950394*	3	3231616-4799670	22	
40		*QOle.BM.crc-LG3-2*	3	*Lu658/Lu3150*	3	24238080-24238427	33	
41		*QOle.BM.crc-LG5*	5	*Lu5-9728492*	15	11375006	131	
42	LIO	*QLio.BM.crc-LG3*	3	*Lu3-3979616/Lu3-5950394*	3	3231616-4799670	22	
43		*QLio.BM.crc-LG6*	6	*Lu2545*	6	8616550-8616919	61	
44	LIN	*QLin.BM.crc-LG5*	5	*Lu5-9728492*	15	11375006	131	
45	IOD	*QIod.BM.crc-LG5*	5	*Lu5-9728492*	15	11375006	131	
46		*QIod.BM.crc-LG6*	6	*Lu6-2260313/Lu6-2330258*	6	2018434-2088579	57	
47	OIL	*QOil.BM.crc-LG8*	8	*Lu8-22516618/Lu3189*	8	16363106-16363334	78	
48	PRO	*QPro.BM.crc-LG11*	11	*Lu11-21716266/Lu52*	11	19594198-19594398	105	
49	CEW	*QCw.BM.crc-LG4*	4	*Lu2031*	4	14489225-14489333	40	
50	STW	*QSw.BM.crc-LG4*	4	*Lu2031*	4	14489225-14489333	40	
51	TSW	*QTsw.BM.crc-LG15*	15	*Lu2010a/Lu2001*	3	20394564-20394673	31	
52	SEB	*QSpb.BM.crc-LG4*	4	*Lu2031*	4	14489225-14489333	40	
53	YLD	*QYld.BM.crc-LG4*	4	*Lu2031*	4	14489225-14489333	40	
54	DTM	*QDm.BM.crc-LG4*	4	*Lu2031*	4	14489225-14489333	40	
55	PLH	*uq.C1–1*		*Lu1_396428*	1	6539309-6539089	3	[18]
56		*uq.C3–1*		*Lu3_693423*	3	25295008-25294801	34	
57		*uq.C4–1*		*Lu4_300701*	4	19453432-19453704	42	
58		*uq.C5–1*		*Lu5_8504*	5	8681823-8682018	45	
59		*uq.C6–1*		*Lu6_639236*	6	2175711-2175911	57	
60		*uq.C8–2*		*Lu8_185009*	7 (4)	6427466-6427621 (6238294-6238449)		
61		*uq.C8–3*		*Lu8_119488*	8	28706-28938	72	
62		*uq.C9–1*		*Lu9_503128*	14	4498680-4498955	122	
63		*uq.C11–1*		*Lu11_557617*	11	1276828-1277143	96	
64		*uq.C11–1*		*Lu11_447048*	11	13338945-13339276	100	
65		*uq.C12–1*		*Lu12_696508*	12	1004697-1004929	108	
66		*uq.C12–1*		*Lu12_163596*	12	351979-352221	106	
67		*uq.C13–1*		*Lu13_367183*	13	8997700-8998007	115	
68		*uq.C14–1*		*Lu14_231853*	14	13485754-13486113	126	
69	TL	*uq.C1–1*		*Lu1_695389*	1	5664124-5664330	2	
70		*uq.C2–2*		*Lu2_597057*	2	22508975-22508683	21	
71		*uq.C5–1*		*Lu5_8504*	5	8681823-8682018	45	
72		*uq.C6–1*		*Lu6_639236*	6	2175711-2175911	57	
73		*uq.C7–1*		*Lu7_781312*	7	18087445-18087733	71	
74		*uq.C8–1*		*Lu8_646184*	8	20045574-20045815	80	
75		*uq.C8–2*		*Lu8_185009*	7 (4)	6427466-6427621 (6238294-6238449)		
76		*uq.C9–2*		*Lu9_618122*	14	3378716-3378969	121	
77		*uq.C12–1*		*Lu12_696508*	12	1004697-1004929	108	
78		*uq.C14–1*		*Lu14_231853*	14	13485754-13486113	126	
79	PLH	*Marker4371*	scaffold156 (LG1)		3	6019156-6019499	24	[22]
80	TL	*Marker747228*	scaffold2786 (LG8)		12	3620608-3620934	110	
81	YLD	*Marker799956*	scaffold319 (LG10)		13	3856362-3856771	114	
82		*Marker770415*	scaffold117 (LG12)		6	11929857-11930253	62	
83		*Marker1073071*	scaffold27 (LG12)		6	8701939-8702324	61	
84	STW	*Marker326151*	scaffold33 (LG5)		8	22241866-22242226	81	
85		*Marker2368217*	scaffold355 (LG15)		10	7140622-7140988	92	
86		*Marker614116*	scaffold355 (LG15)		10	7219061-7219445	93	
87	FY	*Marker2603286*	scaffold156 (LG1)		3	6573623-6574023	27	
88		*Marker1722134*	scaffold127 (LG11)		13	10603161-10603485	116	
89	FC	*Marker1051901*	scaffold680 (LG5)		8	21807786-21808148	81	
90		*Marker1561746*	scaffold376 (LG11)		4	8748431-8748795	36	
91	PLH	*scaffold112_114241*	scaffold112	*scaffold112_114241*	1	18444086	11	[26]
92		*scaffold1491_318496*	scaffold1491	*scaffold1491_318496*	6	14006651	63	
93		*scaffold31_1800846*	scaffold31	*scaffold31_1800846*	3	3929932	22	
94		*scaffold344_309662*	scaffold344	*scaffold344_309662*	1	11008279	6	
95		*scaffold51_1349321*	scaffold51	*scaffold51_1349321*	4	10532424	37	
96		*scaffold59_572553*	scaffold59	*scaffold59_572553*	1	10051709	4	
97		*scaffold156_641874*	scaffold156	*scaffold156_641874*	3	5906791	23	
98		*scaffold147_367986*	scaffold147	*scaffold147_367986*	5	11288517	48	
99		*scaffold859_123972*	scaffold859	*scaffold859_123972*	15	1939372	129	
100	TL	*scaffold297_275113*	scaffold297	*scaffold297_275113*	1	16435852	9	
101		*scaffold361_14957*	scaffold361	*scaffold361_14957*	1	16726904	10	
102		*scaffold273_68457*	scaffold273	*scaffold273_68457*	8	585113	73	
103	NB	*scaffold116_30201*	scaffold116	*scaffold116_30201*	2	9550662	18	
104		*scaffold156_1203677*	scaffold156	*scaffold156_1203677*	3	6468562	26	
105		*scaffold1863_545*	scaffold1863	*scaffold1863_545*	8	1223698	74	
106		*scaffold212_601171*	scaffold212	*scaffold212_601171*	6	6380495	60	
107		*scaffold353_773806*	scaffold353	*scaffold353_773806*	5	16077893	54	
108		*scaffold42_494571*	scaffold42	*scaffold42_494571*	13	15861394	117	
109		*scaffold464_754364*	scaffold464	*scaffold464_754364*	14	15460919	127	
110		*scaffold635_43971*	scaffold635	*scaffold635_43971*	8	22494547	82	
111		*scaffold977_784147*	scaffold977	*scaffold977_784147*	11	18799131	104	
112		*scaffold212_216830*	scaffold212	*scaffold212_216830*	6	5996154	59	
113		*scaffold359_282990*	scaffold359	*scaffold359_282990*	14	6711296	124	
114		*scaffold359_289139*	scaffold359	*scaffold359_289139*	14	6705147	123	
115		*scaffold977_469888*	scaffold977	*scaffold977_469888*	11	18484872	103	
116	FN	*scaffold137_111000*	scaffold137	*scaffold137_111000*	1	11869417	7	
117		*scaffold225_427119*	scaffold225	*scaffold225_427119*	8	15994154	77	
118		*scaffold687_121617*	scaffold687	*scaffold687_121617*	14	16813947	128	
119		*scaffold156_761294*	scaffold156	*scaffold156_761294*	3	6026211	24	
120		*scaffold413_1116527*	scaffold413	*scaffold413_1116527*	4	16914228	41	
121		*scaffold156_1203677*	scaffold156	*scaffold156_1203677*	3	6468562	26	
122		*scaffold413_388319*	scaffold413	*scaffold413_388319*	5	14910709	52	
123		*scaffold687_123666*	scaffold687	*scaffold687_123666*	14	16811898	128	
124	TSW	*scaffold101_354340*	scaffold101	*scaffold101_354340*	3	20942454	32	
125		*scaffold112_184204*	scaffold112	*scaffold112_184204*	1	18514049	11	
126		*scaffold1143_190268*	scaffold1143	*scaffold1143_190268*	1	4375935	1	
127		*scaffold1155_171787*	scaffold1155	*scaffold1155_171787*	15	7690615	130	
128		*scaffold123_1191347*	scaffold123	*scaffold123_1191347*	11	3875819	98	
129		*scaffold1317_154716*	scaffold1317	*scaffold1317_154716*	15	15275145	133	
130		*scaffold132_713877*	scaffold132	*scaffold132_713877*	1	24877317	14	
131		*scaffold1491_58878*	scaffold1491	*scaffold1491_58878*	6	14266269	64	
132		*scaffold15_1207948*	scaffold15	*scaffold15_1207948*	5	16914987	55	
133		*scaffold1519_272169*	scaffold1519	*scaffold1519_272169*	9	1027739	84	
134	FN	*scaffold346-438191*	scaffold346	*scaffold346-438191*	14	1083228	120	[17]
135	TSW	*scaffold43-1111162*	scaffold43	*scaffold43-1111162*	2	21989104	19	
136		*scaffold51-598586*	scaffold51	*scaffold51-598586*	4	11283142	39	
137		*scaffold51-598611*	scaffold51	*scaffold51-598611*	4	11283117	39	
138		*scaffold51-699833*	scaffold51	*scaffold51-699833*	4	11181895	38	
139		*scaffold261-925068*	scaffold261	*scaffold261-925068*	9	6419385	80	
140		*scaffold373-545801*	scaffold373	*scaffold373-545801*	13	17912691	119	
141		*scaffold373-545816*	scaffold373	*scaffold373-545816*	13	17912706	119	
142		*scaffold107-300735*	scaffold107	*scaffold107-300735*	2	22405177	20	
143	PAL	*scaffold59-164258*	scaffold59	*scaffold59-164258*	1	10459958	5	
144	STE	*scaffold11-96400*	scaffold11	*scaffold11-96400*	5	9964973	46	
145		*scaffold11-96569*	scaffold11	*scaffold11-96569*	5	9965142	46	
146	LIO	*scaffold1253-27622*	scaffold1253	*scaffold1253-27622*	9	1922095	85	
147	LIN	*scaffold416-80582*	scaffold416	*scaffold416-80582*	5	13560525	50	
148		*scaffold302-224377*	scaffold302	*scaffold302-224377*	5	13889425	51	
149		*scaffold302-224395*	scaffold302	*scaffold302-224395*	5	13889443	51	
150	FC	*scaffold179-179593*	scaffold179	*scaffold179-179593*	2	2253135	17	
151		*scaffold866-116645*	scaffold866	*scaffold866-116645*	6	1083247	56	
152	PLH	*scaffold344-309662*	scaffold344	*scaffold344-309662*	1	11008279	6	
153		*scaffold59-572553*	scaffold59	*scaffold59-572553*	1	10051709	4	
154	TL	*scaffold297-275113*	scaffold297	*scaffold297-275113*	1	16435852	9	
155		*scaffold297-275131*	scaffold297	*scaffold297-275131*	1	16435834	9	
156		*scaffold361-14957*	scaffold361	*scaffold361-14957*	1	16726904	10	
157	MC	*Lu2-22298066*	2	*Lu2-22298066*	2	22402960	20	[27]
158		*Lu3-25559600*	3	*Lu3-25559600*	3	17645461	29	
159		*Lu3-26033342*	3	*Lu3-26033342*	3	18058033	30	
160		*Lu3-7398487*	3	*Lu3-7398487*	3	6246253	25	
161		*Lu5-3808878*	5	*Lu5-3808878*	5	4087340	44	
162		*Lu7-13225294*	7	*Lu7-13225294*	7	12048040	68	
163		*Lu11-2498303*	11	*Lu11-2498303*	11	2755439	97	
164	HC	*Lu7-6577527*	7	*Lu7-6577527*	7	5834429	67	
165		*Lu10-21552161*	10	*Lu10-21552161*	4	4609469	35	
166		*Lu12-5267706*	12	*Lu12-5267706*	12	5160897	112	
167		*Lu13-2803224*	13	*Lu13-2803224*	13	2764903	113	
168	YLD	*QYLD-Lu4.1*	4	*Lu4-13594936-Lu4-14968389*	4	13593668-14966967	40	[28]
169	OIL	*QOIL-Lu2.1*	2	*Lu2-21913720-Lu2-21913720*	2	21912675	19	
170		*QOIL-Lu5.2*	5	*Lu5-15704607-Lu5-15705039*	5	15703416-15703848	53	
171		*QOIL-Lu6.3*	6	*Lu6-4879632-Lu6-4879632*	6	4879493	58	
172		*QOIL-Lu6.4*	6	*Lu6-13799180-Lu6-13970951*	6	13798861-13970632	63	
173		*QOIL-Lu7.4*	7	*Lu7-14209179-Lu7-14209179*	7	14208772	69	
174		*QOIL-Lu10.5*	10	*Lu10-6517448-Lu10-6517448*	10	6517339	91	
175		*QOIL-Lu12.6*	12	*Lu12-4591214-Lu12-7491405*	12	4591134-7490902	112	
176		*QOIL-Lu15.7*	15	*Lu15-14665900-Lu15-15429055*	15	14665228-15428383	132	
177	PLH	*QPLH-Lu1.1*	1	*Lu1-13887715-Lu1-13930292*	1	13887346-13929923	8	
178		*QPLH-Lu1.2*	1	*Lu1-20012490-Lu1-20012490*	1	20011813	12	
179		*QPLH-Lu4.3*	4	*Lu4-14305982-Lu4-15042104*	4	14304616-15040682	40	
180		*QPLH-Lu13.4*	13	*Lu13-17243884-Lu13-17243884*	13	17242916	118	
181		*QPLH-Lu13.5*	14	*Lu14-2320469-Lu14-2320469*	14	2320188	121	
182	PAL	*QPAL-Lu5.1*	5	*Lu5-12062376-Lu5-12182441*	5	12061283-12181348	49	
183		*QPAL-Lu5.2*	5	*Lu5-13797851-Lu5-15668995*	5	13796740-15667804	51	
184		*QPAL-Lu7.3*	7	*Lu7-624461-Lu7-5423691*	7	624439-5423600	66	
185		*QPAL-Lu11.4*	11	*Lu11-4417685-Lu11-4429424*	11	4417306-4429045	99	
186	IOD	*QIOD-Lu4.1*	4	*Lu4-19909467-Lu4-19909467*	4	19907982	43	
187		*QIOD-Lu7.2*	7	*Lu7-15346458-Lu7-17977459*	7	15346004-17976903	70	
188		*QIOD-Lu12.3*	12	*Lu12-489561-Lu12-2981642*	12	489561-2981562	107	
189	LIN	*QLIN-Lu4.1*	4	*Lu4-19909467-Lu4-19909467*	4	19907982	43	
190		*QLIN-Lu7.2*	7	*Lu7-14540719-Lu7-17977459*	7	14540265-17976903	70	
191		*QLIN-Lu12.3*	12	*Lu12-489561-Lu12-2981642*	12	489561-2981562	107	
192	LIO	*QLIO-Lu4.1*	4	*Lu4-19909467-Lu4-19909467*	4	19907982	43	
193		*QLIO-Lu7.2*	7	*Lu7-14540706-Lu7-17977459*	7	14540252-17976903	70	
194		*QLIO-Lu12.3*	12	*Lu12-489561-Lu12-2981642*	12	489561-2981562	107	
195	DTM	*QDTM-Lu4.1*	4	*Lu4-13171757-Lu4-15042104*	4	13170489-15040682	40	
196		*QDTM-Lu11.2*	11	*Lu11-14768686-Lu11-14768686*	11	14767787	101	
197	STE	*QSTE-Lu9.1*	9	*Lu9-4229230-Lu9-4229230*	9	4229031	87	
198		*QSTE-Lu9.2*	9	*Lu9-20080531-Lu9-21636823*	9	20079433-20654527	90	
199	PRO	*QPRO-Lu15.1*	15	*Lu15-14746288-Lu15-14746310*	15	14745616-14745638	132	
200	OLE	*QOLE-Lu8.1*	8	*Lu8-21782841-Lu8-23527563*	8	21781910-23526575	81	

See Table 1 for additional note.

**Table 4:** Resistant gene analog (RGA) candidates near three QTL for flax powdery mildew resistance.

QTL No.	QTL	Chr	QTL Coordinates (bp)	RGA	Gene Location on chr (bp)	Gene Annotation
12	*QPM-crc-LG1*	1	16920407-18739647	*Lus10026756*	17134471	RLK
				*Lus10026761*	17159664	RLK
				*Lus10026765*	17189168	NBS
				*Lus10009703*	18125241	RLK
13	*QPM-crc-LG7*	7	3817603-3817863	*Lus10023437*	3725947	TM-CC
14	*QPM-crc-LG9*	9	357191-357510	*Lus10001677*	429431	RLK

NBS: nucleotide binding site; RLK: receptor-like protein kinase; TM-CC: transmembrane coiled-coil.

## Supplementary Materials

The following are available online at https://www.mdpi.com/2409-9279/3/2/28/s1. **Table S1**. Information related to the pseudomolecules of 15 chromosomes in the NCBI database. The downloaded sequences from NCBI are used as input for **Program S1**. **Table S2**. Primer sequences of SSR markers for the identified QTL. **Table S3**. Flanking sequences of SNP markers for the identified QTL. **Table S4**. Coordinates of flax scaffold sequences on the most recent release of the chromosome-scale pseudomolecules. This file is used as input for **Program S2**. **Table S5**. Coordinates and annotations of flax protein coding genes on the most recent release of the chromosome-scale pseudomolecules. This file is used as input for **Program S4**. **Table S6**. Coordinates and annotations of flax resistance gene analogs on the recently released chromosome-scale pseudomolecules. This file is used as input for **Program S4**. **Table S7**. Candidate gene prediction of the 200 QTL in Table 3. **Program S1**. A Perl script to prepare a search database of reference sequences for electronic PCR. Program file name: *ProgramS1_prepare_rePCR.pl*. **Program S2**. A Perl script to perform electronic PCR, i.e., map a pair of PCR primer sequences to a reference sequence. Program file name: *ProgramS2_rePCR_pipeline.pl*. **Program S3**. A Perl script to convert coordinates of flax scaffold sequences onto the chromosome-scale pseudomolecules. Program file name: *ProgramS3_convert_scaffold_coordinates_to_pseudochr.pl*. **Program S4**. A Perl script to extract all candidate genes and gene annotation information (protein-coding genes or specifically resistance gene analogs) within a genomic region of a QTL or a marker. Program file name: *ProgramS4_flax_QTL_candidate_gene_scanning.pl* **User guide S1**. A user guide for executions of **Programs S1**, **S2**, **S3**, and **S4**. All programs are also available in the gitHub site: https://github.com/ORDC-Crop-Bioinformatics/Mapping_QTL_in_Flax.
