# Supplementary material for: Mapping Quantitative Trait Loci onto Chromosome-Scale Pseudomolecules in Flax
Source: Methods Protoc. 2020 Apr 4;3(2):28. doi: 10.3390/mps3020028 (PMC7359702; doi:10.3390/mps3020028)
Supplement: Supplementary file 1 [file mps-03-00028-s001.zip › supp_files/program_packages/UserGuide_S1.pdf]

A User Guide for Mapping QTL onto the Most Recent Release of the Chromosome-scale Pseudomolecules in Flax

Frank M. You and Sylvie Cloutier

Ottawa Research and Development Centre, Agriculture and Agri-Food Canada, Ottawa, ON K1A 0C6, Canada

This user guide provides a brief description of the methods with their software tools and database files for mapping QTL onto the most recent release of the chromosome-scale pseudomolecules in flax (You et al. 2018).

In the supplementary files, there is a folder named “program_packages” that contains four Perl scripts (Program_S[1-4]_*.pl), a user guide document (UserGuideS1.doc), and two subfolders “sample_data” and “database_files”. Please copy all scripts and files in the two subfolders to a working folder.

1. Reference sequences

The most recent release of the flax pseudomolecules (You et al. 2016) includes 15 sequences corresponding to 15 flax chromosomes. Their accession numbers in NCBI and sequence lengths are listed in the following table:

Sequences of 15 chromosomes in the NCBI database.

Chromosome	NCBI accession	Length of sequence (bp)
Lu1	CP027619	29,425,369
Lu2	CP027626	25,730,386
Lu3	CP027627	26,636,119
Lu4	CP027628	19,927,942
Lu5	CP027629	17,699,757
Lu6	CP027630	18,078,158
Lu7	CP027631	18,299,719
Lu8	CP027632	23,785,339
Lu9	CP027633	22,091,576
Lu10	CP027620	18,203,127
Lu11	CP027621	19,887,771
Lu12	CP027622	20,889,232
Lu13	CP027623	20,483,506
Lu14	CP027624	19,392,306
Lu15	CP027625	15,636,771
Total		316,167,078

These sequences can be downloaded directly from the NCBI nucleotide databases by searching individual accession number, for example, at https://www.ncbi.nlm.nih.gov/nuccore/CP027619, or by using NCBI Batch Entrez for batch download at https://www.ncbi.nlm.nih.gov/sites/batchentrez. All sequences can be saved in a fasta file.

If you only need to convert coordinates of SNPs from the scaffolds based reference sequences onto the most recent release of the chromosome-scale pseudomolecules, you do not need to download these sequence. These sequences are required only when you map PCR based markers to the flax pseudomolecules.

2. Mapping PCR based markers to the most recent release of the chromosome-scale pseudomolecules

The electronic PCR (e-PCR) method is used to map paired PCR primers onto the flax pseudomolecules (Schuler, 1997). The source code for the e-PCR program is freely available (ftp://ncbi.nlm.nih.gov/pub/schuler/e-PCR/).

Two executive programs, “fahash” and “re-PCR”, are included in the e-PCR program package. You need to download the source code to compile it and then place two executable files into the same working directory. The two executable programs can be also saved in any other folder but a path pointing to them must be correctly set so that the Perl scripts can access them.

Step 1. Create a search database

Usage:
```
perl ProgramS1_prepare_rePCR_data.pl
  -i fasta file name of the reference sequence (* is allowed)
  -s genome name as file name prefix
  -m maximum number of sequences for each database (Default:5000)
```

Default parameters for the “fahash” program are used in the ProgramS1_prepare_rePCR_data.pl script:
```
$cmd = "fahash -b $genome_hash -w 12 -f3 $genome_map";
```

Two output files “*.famap” and “*.hash” will be generated in this step.

Step 2. Mapping PCR markers to the reference genome

Usage:
```
perl ProgramS2_rePCR_pipeline.pl
     -p primer file (required)
     -d reference genome hash file (required, generated from
ProgramS1_prepare_rePCR_data.pl, both *.famap and *.hash must be available)
     -m number of mismatches (default: 0)
     -g number of gaps (default: 0)
```

Example:

Step 1:
Flax pseudomolecule sequences are saved in a file in fasta format, e.g., **flax_pseudomolecules.fasta**

```
perl ProgramS1_prepare_rePCR_data.pl -i flax_pseudomolecules.fasta -s
flax_new_pseudo
```

Two output files will be generated in this step:
`flax_new_pseudo_1.famap`

`flax_new_pseudo_1.hash`

Step 2:

```
perl ProgramS2_rePCR_pipeline.pl -p  program_S2_sample_marker_data.txt -d
flax_new_pseudo_1.hash
```

A sample marker file "program_S2_sample_marker_data.txt" is included in the program package. This file contains a header line with four columns separated by a tab key (\t). The last column is the amplicon size range of the PCR marker. It must have the following format:

Primer ID	Forward Primer	Reverse Primer	Size range
Lu2164	GCATGATCGTTACTTTAGGATGC	AATGACGCCATCTTTTGTCC	50-1500
Lu2183	CTTCATGCAGTCCGTTTTTACA	CAGTTCGTAGTTTACTTGGTGCAG	50-1500
Lu2532	GGATAGAAGCTCACCGATGC	TTCAGAGCACCAGCAGAAAA	50-1500
Lu2545	TGCTTTGCTAATTTATTATGGTGAG	ATGGTAGCTGGTGGGTGAAC	50-1500
Lu2555	TCCCGCTTTTAATGGTGTTC	AATTGGAAGCTCGATTCACG	50-1500
Lu2560	CGTGGCTACTAGCAATGTGG	TCCTCATGTTCATTGCTTGC	50-1500
Lu2564	TTTCAGCTTCGATTGAGACG	ATCCGTCGAGGTAACAGTCC	50-1500

The "`flax_new_pseudo_1.hash`" and "`flax_new_pseudo_1.famap`" generated from Step 1 must be in the same working directory.

An output file "`program_S2_sample_marker_data.txt_primer_rePCR_results.txt`" will be generated:

```
#- sts   seq  strand  from      to        mism  gaps  act_len/exp_len
Lu2164     1  -       22948222  22948580     0     0  359/50-1500
Lu2183     1  -       26435050  26435329     0     0  280/50-1500
Lu2555     6  +       14948801  14948986     0     0  186/50-1500
Lu2560     6  -       13553559  13553779     0     0  221/50-1500
Lu2564     6  -       13620999  13621234     0     0  236/50-1500
Lu2532     7  -         661757    662020     0     0  264/50-1500
#-
Done
```

3. **Mapping SNPs on scaffold reference sequences to the most recent release of the chromosome-scale pseudomolecules**

Usage:

```
perl ProgramS3_convert_scaffold_coordinates_to_pseudochr.pl
     -m scaffold to pseudomolecule mapping file. Table S4 must be used.
     -d scaffold coordinate data file which must have three column: marker name,
scaffold IDs and coordinates
```

The "`program_S3_sample_marker_data.txt`" is a sample marker data file that must contain three columns separated by a tab key (\t):

Marker	Scaffold ID	Coordinate_on_scaffold
scaffold112_114241	scaffold112	114241
scaffold1491_318496	scaffold1491	318496
scaffold31_1800846	scaffold31	1800846
scaffold344_309662	scaffold344	309662
scaffold51_1349321	scaffold51	1349321
scaffold59_572553	scaffold59	572553
scaffold156_641874	scaffold156	641874
scaffold147_367986	scaffold147	367986
scaffold859_123972	scaffold859	123972
scaffold297_275113	scaffold297	275113
scaffold361_14957	scaffold361	14957
scaffold273_68457	scaffold273	68457

The "`TableS4_flax_scaffolds_corrdinates_in_new_pseudomolecules.txt`" is a database file that contains accurate information for mapping scaffold sequences to the pseudomolecules. This file is provided in the program package.

Example:

```
perl ProgramS3_convert_scaffold_coordinates_to_pseudochr.pl -m
TableS4_flax_scaffolds_corrdinates_in_new_pseudomolecules.txt -d
program_S3_sample_marker_data.txt
```

A "`program_S3_sample_marker_data.txt.converted.txt`" will be generated:

Marker	Scaffold ID	Coordinate_on_scaffold	Chr	New_Chr_Coord
scaffold112_114241	scaffold112	114241	1	18444086
scaffold1491_318496	scaffold1491	318496	6	14006651
scaffold31_1800846	scaffold31	1800846	3	3929932
scaffold344_309662	scaffold344	309662	1	11008279
scaffold51_1349321	scaffold51	1349321	4	10532424
scaffold59_572553	scaffold59	572553	1	10051709
scaffold156_641874	scaffold156	641874	3	5906791
scaffold147_367986	scaffold147	367986	5	11288517
scaffold859_123972	scaffold859	123972	15	1939372
scaffold297_275113	scaffold297	275113	1	16435852
scaffold361_14957	scaffold361	14957	1	16726904
scaffold273_68457	scaffold273	68457	8	585113

The last two columns are the converted results, including chromosome numbers and new coordinates on chromosomes.

4. Candidate Gene Scanning of QTL

Usage:

```
perl ProgramS4_flax_QTL_candidate_gene_scanning.pl
     -q QTL file
     -d gene annotation file
     -w upstream or downstream window size (bp) (default: 100000 bp)
```

A sample data file for QTL data "program_S4_sample_qtl_data.txt" is provided in the program package. This file must have four columns separated by a tab key (\t):

```
Trait    QTL            Chr   Coord_start    Coord_end
PM       QPM-crc-LG1    1     16920407       18739647
PM       QPM-crc-LG7    7     3817603        3817863
PM       QPM-crc-LG9    9     357191         357510
```

The gene annotation files "TableS6_flax_RGA_coords.txt" and "TableS5_flax_all_genes_coords.txt" are two database files that contain coordinates of all resistance gene analogs (RGAs) or all protein coding genes on the pseudomolecules and their functional annotation information. Each of them can be used for a different purpose (scanning RGAs or all genes). These two files are provided in the program package.

The upstream or downstream window size of the QTL position can be specified. The default value is 100000 bp (i.e. 100 kb). That means that all genes/RGAs within a total of 200 kb window of both upstream and downstream of a QTL on a chromosome will be scanned. You can input a different value for the window size.

Example 1: scan resistance gene analogs within a 200 kb window covering upstream and downstream of the QTL position (default)

```
perl ProgramS4_flax_QTL_candidate_gene_scanning.pl -q
program_S4_sample_qtl_data.txt -d TableS6_flax_RGA_coords.txt
```

A result file "program_S4_sample_qtl_data.txt_gene_annotations.txt" will be generated in the following format:

Trait	QTL	Chr	Coord_st	Coord_er	chr	ID	start	end	strand	type	scaffold	refined scaff
PM	QPM-crc-LG1	1	1.7E+07	1.9E+07	1	Lus10026756	17134471	17137673	+	RLK	scaffold361	scaffold361
PM	QPM-crc-LG1	1	1.7E+07	1.9E+07	1	Lus10026761	17159664	17161647	+	RLK	scaffold361	scaffold361
PM	QPM-crc-LG1	1	1.7E+07	1.9E+07	1	Lus10026765	17189168	17189470	-	NBS	scaffold361	scaffold361
PM	QPM-crc-LG1	1	1.7E+07	1.9E+07	1	Lus10009703	18125241	18127926	+	RLK	scaffold455	scaffold455
PM	QPM-crc-LG7	7	3817603	3817863	7	Lus10023437	3725947	3732607	+	TM-CC	scaffold1216	scaffold1216
PM	QPM-crc-LG9	9	357191	357510	9	Lus10001677	429431	436772	-	RLK	scaffold2739	scaffold2739

Example 2: scan all protein coding genes within a 200 kb window covering upstream and downstream of the QTL position (default)

```
perl ProgramS4_flax_QTL_candidate_gene_scanning.pl -q
program_S4_sample_qtl_data.txt -d TableS5_flax_all_genes_coords.txt
```

A result file with the same file name "`program_S4_sample_qtl_data.txt_gene_annotations.txt`" will be generated in the following format that slightly different from Example 1 (only part of results are shown):

Trait	QTL	Chr	Coord_star	Coord_end	chr	ID	start	end	strand	functional annotation	scaffold	refined scaf	arabidopsis	gene names	dummy
PM	QPM-crc-LG1	1	16920407	18739647	1	Lus10018097	18332046	18334006	+	Lipase class 3-related protein	scaffold112	scaffold112	AT5G24230.1		1
PM	QPM-crc-LG1	1	16920407	18739647	1	Lus10018098	18338780	18340866	+	GRAS family transcription factor family protein	scaffold112	scaffold112	AT2G01570.1	RGA,RGA1	1
PM	QPM-crc-LG1	1	16920407	18739647	1	Lus10018099	18362462	18364039	-	GRAS family transcription factor family protein	scaffold112	scaffold112	AT2G01570.1	RGA,RGA1	1
PM	QPM-crc-LG1	1	16920407	18739647	1	Lus10018100	18395486	18397366	-	GRAS family transcription factor family protein	scaffold112	scaffold112	AT2G01570.1	RGA,RGA1	1
PM	QPM-crc-LG1	1	16920407	18739647	1	Lus10018101	18399363	18400709	-	GRAS family transcription factor family protein	scaffold112	scaffold112	AT2G01570.1	RGA,RGA1	1
PM	QPM-crc-LG1	1	16920407	18739647	1	Lus10018102	18406876	18410238	+	Glycosyl hydrolase family protein	scaffold112	scaffold112	AT5G20950.1		1
……															

References

You, F.M.; Xiao, J.; Li, P.; Yao, Z.; Jia, G.; He, L.; Zhu, T.; Luo, M.-C.; Wang, X.; Deyholos, M.K., *et al*. Chromosome-scale pseudomolecules refined by optical, physical, and genetic maps in flax. *Plant J*. **2018,** 95, (2), 371-384.

Schuler, G.D. Sequence mapping by electronic PCR. *Genome Res*. **1997,** 7, (5), 541-550.
