# Supplementary material for: Mapping Quantitative Trait Loci onto Chromosome-Scale Pseudomolecules in Flax
Source: Methods Protoc. 2020 Apr 4;3(2):28. doi: 10.3390/mps3020028 (PMC7359702; doi:10.3390/mps3020028)
Supplement: Supplementary file 1 [file mps-03-00028-s001.zip › supp_files/supp_tables/TableS1_chromosome_sizes.docx]

**Table S1.** Sequences of 15 chromosomes in the NCBI database.

| Chromosome | NCBI accession | Length of sequence (bp) |
| --- | --- | --- |
| Lu1 | CP027619 | 29,425,369 |
| Lu2 | CP027626 | 25,730,386 |
| Lu3 | CP027627 | 26,636,119 |
| Lu4 | CP027628 | 19,927,942 |
| Lu5 | CP027629 | 17,699,757 |
| Lu6 | CP027630 | 18,078,158 |
| Lu7 | CP027631 | 18,299,719 |
| Lu8 | CP027632 | 23,785,339 |
| Lu9 | CP027633 | 22,091,576 |
| Lu10 | CP027620 | 18,203,127 |
| Lu11 | CP027621 | 19,887,771 |
| Lu12 | CP027622 | 20,889,232 |
| Lu13 | CP027623 | 20,483,506 |
| Lu14 | CP027624 | 19,392,306 |
| Lu15 | CP027625 | 15,636,771 |
| Total |  | 316,167,078 |
